# Automated Deauville Score computation from baseline [¹⁸F]FDG PET/CT predicts progression-free survival in multiple myeloma: a radiogenomic framework

**DOI:** 10.1007/s00259-026-08103-x

**Published:** 2026-07-30

**Authors:** Sara Peluso, Stefano Polizzi, Lisa Pagnini, Martina Tarozzi, Ettore Rocchi, Valentino Dragonetti, Vincenza Solli, Daniele Dall’Olio, Marco Talarico, Carolina Terragna, Claudia Sala, Elena Zamagni, Stefano Fanti, Cristina Nanni, Gastone Castellani

**Affiliations:** 1 Department of Medical and Surgical Sciences, University of Bologna, Bologna, 40138 Italy; 2 IRCCS Azienda Ospedaliero Universitaria di Bologna, Bologna, 40138 Italy; 3 Data Science and Bioinformatics Laboratory, IRCCS Istituto delle Scienze Neurologiche di Bologna, Bologna, 40139 Italy; 4 Nuclear Medicine, IRCCS Azienda Ospedaliero-Universitaria di Bologna, Bologna, Italy; 5 Division of Nuclear Medicine, IEO European Institute of Oncology IRCCS, Milan, Italy; 6 IRCCS Azienda Ospedaliero-Universitaria di Bologna, Istituto di Ematologia “Seràgnoli”, Bologna, Italy

**Keywords:** FDG PET/CT, Deauville Score, Multiple myeloma, Quantitative imaging, Progression-free survival, Radiogenomics

## Abstract

**Purpose:**

Deauville Score (DS) assessment from [^18^F]FDG PET/CT in multiple myeloma (MM) relies on visual interpretation, limiting reproducibility. This study aimed to develop an automated pipeline for standardised DS computation, evaluate the prognostic value of DS at five anatomical sites, and predict progression-free survival (PFS) by integrating DS with copy number alterations (CNA) and clinical variables.

**Methods:**

A retrospective cohort of 165 newly diagnosed MM patients with baseline FDG PET/CT, CNA profiling, and blood tests was analysed. An automated pipeline computed DS fully automatically for vertebral bone marrow (BM) and long bones (LB), and semi-automatically for focal (FL), paramedullary (PM), and extramedullary (EM) lesions. DS were subdivided into absent (1), low (2–3), and high (4–5) groups and compared via log-rank test. A penalised Cox model with 12 covariates (five DS, three CNAs, haemoglobin, platelet count, age, sex) was evaluated via nested cross-validation for PFS prediction. For inference on individual prognostic contributions, an unpenalised multivariable Cox model was fitted on the full cohort.

**Results:**

In univariate analyses, high LB, PM and EM DS were significantly associated with shorter PFS. The penalised Cox model achieved a C-index of 0.710 [95% CI: 0.689–0.732] in predicting the risk of progression. In the multivariable analysis, age, haemoglobin, BM DS, PM DS and amp(1q) were identified as independent prognostic factors.

**Conclusion:**

Automated DS computation from baseline FDG PET/CT is feasible and, combined with genomic and clinical data, enables a reproducible multimodal approach to prognostic stratification in MM. The pipeline for DS computation is publicly available as an open-source tool (autoDS-PET) at https://github.com/Sara-Peluso/autoDS-PET.

**Supplementary Information:**

The online version contains supplementary material available at https://doi.org/10.1007/s00259-026-08103-x.

## Introduction

Multiple myeloma (MM) is a haematological malignancy of plasma cells accounting for an estimated 187,774 cases and 121,252 deaths worldwide in 2022 [1]. MM is biologically and clinically heterogeneous, with diverse genomic alterations, bone marrow (BM) microenvironment changes, and variable imaging patterns producing a broad spectrum of clinical outcomes.

[^18^F]-fluorodeoxyglucose positron emission tomography/computed tomography ([^18^F]FDG PET/CT) captures distinct aspects of MM by combining functional PET assessment with morphological CT evaluation of underlying osteolytic bone destruction. The International Myeloma Working Group (IMWG) recommends [^18^F]FDG PET/CT both at staging and after therapy to evaluate prognosis and treatment response [2]. In addition to disease metabolic activity, it aids detection of focal lesions, diffuse marrow infiltration, and extramedullary sites of proliferating plasma cells [3, 4].

The absence of standardised imaging criteria hinders wider use of [^18^F]FDG PET/CT, resulting in non-harmonised interpretation and lack of reproducibility across studies. Various criteria have been proposed, including maximum Standardised Uptake Value (SUVmax)-based analyses, volumetric metrics such as Metabolic Tumour Volume (MTV), or Total Lesion Glycolysis (TLG) [5, 6]. A group of MM experts defined a set of visual descriptive criteria (Italian Myeloma criteria for PET Use, IMPeTUs) [7, 8], based on five-point Deauville Score (DS) scale, originally introduced for FDG PET evaluation in lymphoma [9].

For each target Region Of Interest (ROI), DS is computed by comparing its SUV with physiological reference organs’ uptake (mediastinal blood pool, MBP; liver). In lymphoma, DS scores the ROI identified as the highest uptake in a site of initial disease, if present [9]. In MM, DS is applied to vertebral BM, hottest focal bone lesion (FL), hottest paramedullary lesion (PM), and hottest extramedullary lesion (EM) [8]. The present work extends the IMPeTUs framework by introducing a fifth ROI encompassing femoral and humeral diaphyses (long bones, LB) to capture appendicular BM involvement.

Recent studies have explored automated FDG PET/CT quantification in MM. Sachpekidis et al. developed an AI-based tool showing that automated MTV and TLG predict survival in newly diagnosed MM (NDMM) [10, 11]; Jamet et al. demonstrated the prognostic value of medullary total tumour volume [12]. Other groups have applied radiomics and deep learning to extract prognostic features [13–15]; however, these approaches either produce continuous metrics lacking a standardised clinical interpretation framework, or rely on high-dimensional, non-interpretable feature sets. These limitations are barriers to the clinical translation of radiomics [16]; furthermore, no study provides site-specific scores aligned with established reporting frameworks like IMPeTUs [17].

At the genomic level, MM is marked by chromosomal instability with copy number alterations (CNA) like amp(1q), del(13q), and del(17p), occurring in approximately 35–40%, 40–50%, and 5–12% of NDMM cases, respectively [18–20]; these alterations adversely impact Progression Free Survival (PFS) [21–23]. Notably, amp(1q) and del(13q) frequently co-occur and their co-occurrence has been associated with adverse clinical features and shortened survival [21, 24]. Routine haematologic indices provide complementary clinical insight: anaemia reflects BM infiltration; thrombocytopenia indicates compromised haematopoiesis. Both are well-established adverse prognostic factors that, when combined with lytic bone lesions, stratify patients into distinct prognostic groups [25].

Imaging, genomic, and clinical variables capture complementary aspects of MM, and the heterogeneity of MM calls for a multimodal approach to improve prognostic accuracy beyond any single modality. Radiogenomic approaches combining imaging with molecular data have demonstrated improved prognostic performance compared to single-modality approaches across multiple malignancies [26], supporting the rationale for integrating automated DS with genomic and clinical variables in MM.

**Fig. 1:**
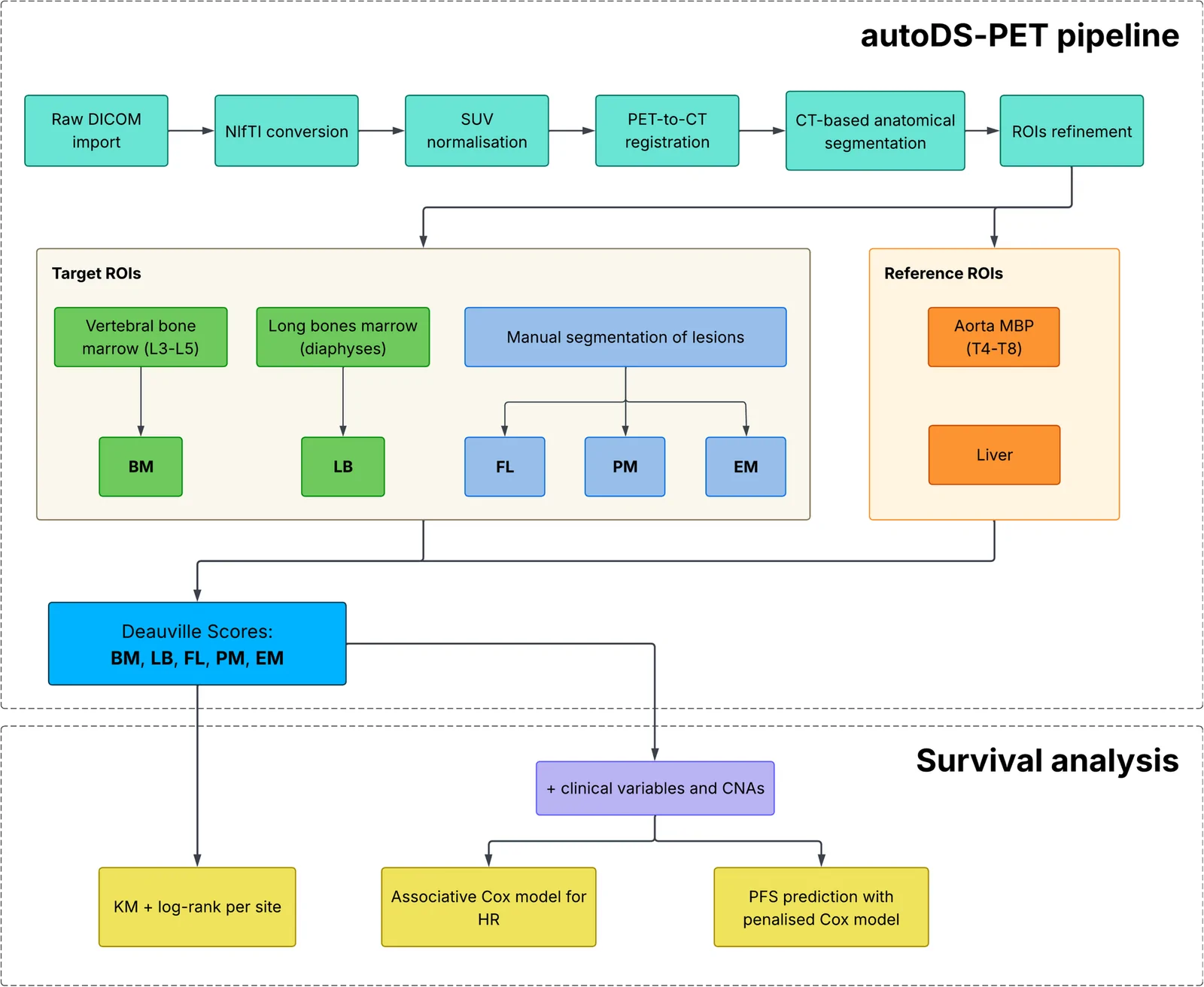
Study workflow. Upper: autoDS-PET pipeline for automated Deauville Score computation from [^18^F]FDG PET/CT. Target ROIs (vertebral bone marrow, long bones) and reference ROIs (mediastinal blood pool, liver) are computed automatically; focal (FL), paramedullary (PM), and extramedullary (EM) lesion masks are manually delineated. SUV extraction and DS assignment are performed automatically. Lower: survival analysis framework – Kaplan-Meier analysis of site-specific DS, and DS combined with clinical and CNA covariates for predictive and associative PFS analyses

The primary aims of this retrospective study are to develop an open-source pipeline for standardised DS computation from baseline [^18^F]FDG PET/CT in NDMM and to evaluate its potential for PFS prediction and risk stratification at diagnosis. The pipeline runs fully automatically for diffuse marrow assessment (vertebral and long bones marrow) and semi-automatically for lesion-level analysis (FL, PM, EM) when expert segmentations are provided. To assess the clinical relevance of automated DS, we compared PFS between low (1–3) and high (4–5) DS groups at each site, then integrated DS with cytogenetic and clinical variables in a penalised Cox model for PFS prediction and identified prognostic factors through a multivariable associative analysis. Analogous overall survival (OS) analyses are reported in Supplementary Material. The study workflow is illustrated in Fig. 1.

## Materials and methods

### Patients

This retrospective monocentric study included 165 NDMM patients diagnosed between 2007 and 2021 at IRCCS Azienda Ospedaliero-Universitaria di Bologna (Bologna, Italy) according to IMWG criteria. All patients had active MM requiring treatment at the time of inclusion and provided written informed consent.

Inclusion criteria were: (i) availability of baseline [^18^F]FDG PET/CT, acquired before starting any myeloma-directed therapy; (ii) availability of CNA profiling at diagnosis; (iii) availability of routine blood test results at diagnosis. No formal exclusion criteria were applied beyond the above requirements.

Demographic and clinical variables included age, biological sex, haemoglobin level (HB, g/dL), and platelet count (PLT, 10^9^/L), together with three CNAs: amp(1q), del(13q), and del(17p), included among those recommended in the IMWG molecular classification as recurrent and clinically relevant CNAs in MM [27]; IgH translocations were not systematically available across the full cohort and were therefore not included (analyses on the subgroup with high-risk translocations reported in Supplementary Material). A full cohort description is provided in Table 1. PFS was defined as the time from treatment initiation to disease progression or death by any cause, with patients alive and progression-free censored at their last follow-up. Progression was assessed through routine clinical follow-up encompassing biochemical, clinical, and imaging criteria. The most systematically documented markers were biochemical (increasing serum free light chains and/or monoclonal protein levels). Median follow-up was estimated using the reverse Kaplan-Meier (KM) method [28].

**Table 1:** Baseline demographic, clinical, CNA, and survival characteristics of the study cohort (*n* = 165)

Patients’ characteristics	Values	*n* (%)
Biological sex	M	99 (60%)
	F	66 (40%)
Age (years)	Median	64
	IQR	[58, 69]
	≥ 65 y.o.	77 (47%)
R-ISS stage	I	39 (24%)
	II	72 (44%)
	III	20 (12%)
	Unknown	34 (20%)
Haemoglobin	< 10 g/dL^a^	52 (31%)
Platelet count	< 150 × 10^9^/L^b^	24 (14%)
amp(1q)	1	60 (36%)
del(13q)	1	70 (42%)
del(17p)	1	11 (7%)
Autologous stem cell transplant	1	115
	0	42
	Unknown	8
Induction therapy	IMiD	11
	PI	35
	Anti-CD38 – IMiD	2
	Anti-CD38 – PI	12
	Anti-CD38 – PI – IMiD	6
	PI – IMiD	99
PFS event	0	66 (40%)
	1	99 (60%)
PFS time (months)	Median	30
	IQR	[19, 44]
OS event	0	117 (70%)
	1	48 (30%)
Follow-up time (months)	Median	50
	IQR	[37, 60]

Continuous variables reported as median and interquartile range (IQR); categorical variables as count and percentage. *R-ISS* Revised International Staging System, *HB* haemoglobin, *PLT* platelet count, *PI* Proteasome inhibitor, *IMiD* immunomodulatory drug, *PFS* progression-free survival, *OS* overall survival. ^a^ Anaemia threshold: HB < 10 g/dL [29]. ^b^ Thrombocytopenia threshold: PLT < 150 × 109/L [25]

### PET/CT data acquisition

Baseline [^18^F]FDG PET/CT images were acquired using four GE Medical Systems scanners with a median uptake time of 78 min (IQR: 65–88 min). All PET images underwent scatter correction and decay correction with respect to the acquisition start time and were reconstructed using iterative OSEM-based algorithms. PET matrix size was 128 × 128 (*n* = 93) or 256 × 256 (*n* = 72); CT images were reconstructed at 512 × 512. Detailed acquisition parameters are reported in Table 2.

**Table 2:** [^18^F]FDG PET/CT acquisition parameters

Acquisition parameters	Values	*n*
Scanner model	Discovery STE	75
	Discovery 710	45
	Discovery MI	27
	Discovery LS	18
CT parameters		
kVp	120	122
	100	41
	140	2
Pixel spacing (mm)	1.37	102
	0.98	63
Slice thickness (mm)	3.75	147
	5.0	18
PET parameters		
Pixel spacing (mm)	5.47	75
	2.73	72
	3.91	18
Slice thickness (mm)	3.27	120
	2.78	27
	4.25	18

### PET/CT data analysis

PET/CT images were processed using *autoDS-PET*, a dedicated open-source Python pipeline for automatic and semi-automatic DS computation (see Code availability). DICOM images were first converted to NIfTI format using SimpleITK (v2.5.2) [30]. The pipeline comprises: (1) SUV normalisation; (2) intra-patient PET-to-CT registration; (3) CT-based anatomical segmentation; (4) ROIs refinement via morphological operations; (5) SUV extraction; (6) DS assignment.

### SUV normalisation

Raw PET activity concentrations (Bq/mL) were converted to standardised uptake values normalised by body weight (SUVbw) using the standard formula, accounting for injected dose, patient body weight, and ^18^F decay correction to the acquisition start time.

### Intra-patient PET-to-CT registration

PET SUVbw images were rigidly registered to the corresponding CT images using SimpleElastix, with Advanced Mattes Mutual Information as similarity metric [31]. The PET image was resampled onto the CT grid to ensure identical spatial geometry.

### CT-based anatomical segmentation

Segmentation of anatomical ROIs was performed on CT images using TotalSegmentator [32], an open-source, externally-validated deep-learning tool providing pre-trained models for 117 anatomical structures, built on the nnU-Net architecture [33]. Segmented structures included vertebrae (with a separate vertebral bodies mask), aorta, heart, liver, femurs, and humeri.

### ROIs refinement

TotalSegmentator-derived ROIs were refined via image processing operations using SimpleITK. All morphological operations (erosion, dilation) were carried out in physical space using signed Euclidean distance transforms for robustness to anisotropic voxel spacing and variations in spatial resolution across the dataset.

#### Bone marrow ROI

L3, L4, and L5 vertebral masks were extracted from TotalSegmentator multilabel segmentation and combined via logical union. This mask was then intersected with the separately obtained binary vertebral bodies segmentation to isolate the vertebral body compartment, excluding posterior elements. The resulting mask was eroded by 3 mm to retain cancellous bone hosting BM. The erosion margin conservatively exceeds the reported lumbar cortical shell thickness ranging from 0.3 mm in histomorphometric studies [34] up to 1.5 mm in micro-CT assessments [35], and accounts for partial-volume effects.

#### Mediastinal blood pool ROI

The aorta mask was restricted to the axial extent of T4-T8 vertebral slab through slice-wise gating. This procedure was devised to automatically select ascending aorta, aortic arch and the proximal tract of thoracic aorta (European Association of Nuclear Medicine, EANM, guidelines [36]). Cardiac spill-in was mitigated by dilating heart segmentation by 6 mm and subtracting it from the aorta. The resulting mask was eroded by 4 mm to exclude vessel walls and obtain an intraluminal ROI. When multiple disconnected components remained, only the largest one was retained.

#### Liver ROI

The liver mask was reduced to its largest connected component; holes were filled using flood-fill algorithm with full connectivity. The filled mask was eroded by 10 mm to exclude subcapsular regions and partial-volume effects, yielding a homogeneous parenchymal reference region in line with EANM recommendations.

#### Long bones ROI

Left and right femur and humerus masks were individually extracted. Each mask was cropped to the central 60% of its axial extent to approximate the diaphysis, usually not exhibiting FDG uptake but potentially presenting pathological infiltration by myeloma cells, excluding the epiphysis which may present aspecific mild medullary uptake. Each cropped mask was then eroded (5 mm for femora, 4 mm for humeri) to exclude the cortical shell (reported diaphyseal thickness: 1–3 mm in proximal femur, increasing in mid-diaphysis, 3–4 mm in humerus [37, 38]) and adjacent partial-volume effects. The smaller humeral margin preserved sufficient medullary voxels considering the reduced cross-sectional area of humeri. The four eroded diaphyseal masks were combined into a single long bones ROI.

#### Lesion ROIs

FL, PM, and EM lesion masks were manually segmented by a nuclear medicine physician on the fused PET/CT images. When multiple lesions were present at a given site, the lesion with the highest FDG uptake was selected for DS assignment.

### SUV extraction

Summary SUVbw statistics were extracted for each ROI: 95th percentile for BM and LB, providing robustness to focal outliers, and maximum SUVbw for FL, PM, and EM lesions, where peak uptake is clinically meaningful unlike diffuse marrow uptake in BM and LB. Voxel-wise median SUVbw was used for reference ROIs (MBP, liver), offering a robust estimate unaffected by partial-volume artifacts.

### Deauville Score assignment

For each target ROI, DS was assigned by comparing target uptake against reference ROIs uptake. Table 3 reports DS five-point scale with original and computational definitions. For BM and LB, DS = 1 was never assigned given the always-present physiological FDG uptake [39]. FL, PM, and EM may receive DS = 1, indicating no detectable lesion.

**Table 3:** Deauville Score scale: original definition and computational criteria for automated DS assignment. ROI uptake: 95th percentile of SUVbw for BM and LB; maximum SUVbw for FL, PM, and EM. MBP and liver uptake: median SUVbw within reference ROIs

DS value	Original definition	Computational definition
1	No uptake	No uptake
2	Slight uptake, equal to or below blood pool (mediastinum)	ROI uptake ≤ MBP uptake
3	Uptake above mediastinal, but below or equal to uptake in the liver	MBP uptake < ROI uptake ≤ liver uptake
4	Moderately increased uptake compared to liver	Liver uptake < ROI uptake ≤ 2× liver uptake
5	Markedly increased uptake compared to liver	ROI uptake > 2× liver uptake

### Survival analysis

PFS was selected as primary endpoint as it most directly captures the prognostic impact of baseline disease characteristics [40]. Analogous predictive and associative analyses were performed for OS (Supplementary Material).

#### DS subgrouping and Kaplan-Meier analysis

At each site, DS scale was subdivided into absent (DS 1, i.e. compartment not scored), low (DS 2–3), and high (DS 4–5) subgroups [41]; their association with PFS was evaluated using KM method with log-rank test (significance level: α = 0.05).

#### Predictive analysis

A multivariable Cox proportional hazards model with L2 penalisation was used to predict PFS in a nested cross-validation (CV) framework to ensure robust and unbiased performance estimation [42]. The model included 12 covariates and an interaction term: five DS, three CNAs, an interaction term between amp(1q) and del(13q), HB, PLT, age, and sex. The nested CV framework was chosen to simulate the clinical scenario in which a model trained on existing data is applied to stratify previously unseen patients at diagnosis. The inner loop (5-fold CV) tuned the regularisation parameter over {0, 0.01, 0.1, 1, 10, 100} using Uno’s concordance index (C-index) [43]; the outer loop (10-fold CV, 5 repetitions) evaluated performance. Both CVs were stratified by PFS event, and continuous and ordinal variables were standardised within the CV pipeline. Discrimination was quantified using Uno’s C-index, preferred over Harrell’s as it corrects for censoring differences between training and test folds via Inverse Probability of Censoring Weighting (IPCW). Calibration was assessed using the integrated Brier score (IBS) computed over 100 equally spaced timepoints within the IPCW-valid interval. 95% confidence intervals (CI) for both metrics were obtained by bootstrap resampling (10,000 iterations) of per-fold estimates.

#### Associative analysis

To identify individual prognostic factors, an unpenalised multivariable Cox model with the same 13 covariates was fitted on the full cohort; no penalisation was applied to preserve interpretable hazard ratios (HR) estimates. Multicollinearity was assessed using the Generalised Variance Inflation Factor (GVIF) [44], and proportional hazards assumption using a global Grambsch-Therneau test on scaled Schoenfeld residuals [45]. HR with 95% CI and p-values were computed for each covariate. A bootstrap procedure (2000 resamples) was applied as stability analysis of HR estimates.

#### Software

Statistical analyses were performed with Python packages lifelines (v0.30.0) [46], scikit-survival (v0.26.0) [47] and scikit-learn (v1.7.2) [48].

## Results

### Automated and semi-automated DS computation

**Fig. 2:**
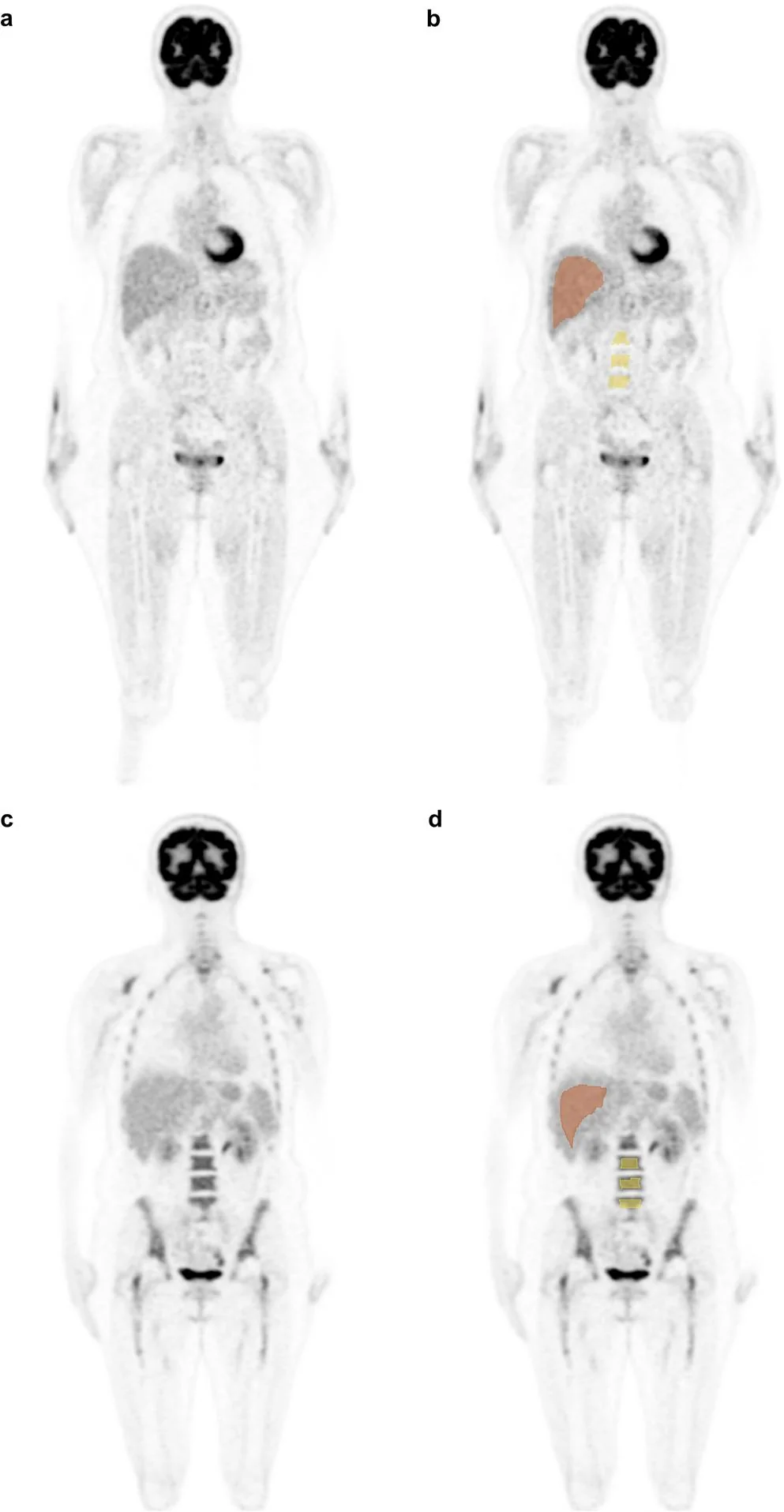
Coronal PET images of a low BM DS patient (DS 2–3; **a**,** b**) and a high BM DS patient (DS 4–5; **c**,** d**), shown without (**a**,** c**) and with (**b**,** d**) eroded liver (orange) and L3-L5 vertebral body (yellow) masks

The automated pipeline ran successfully on all 165 patients; no case required manual intervention or exclusion. Figure 2 shows representative coronal PET image for a low BM DS patient (a, b) and a high BM DS patient (c, d), with and without refined segmentations of liver and L3-L5 vertebral body ROIs overlaid.

**Fig. 3:**
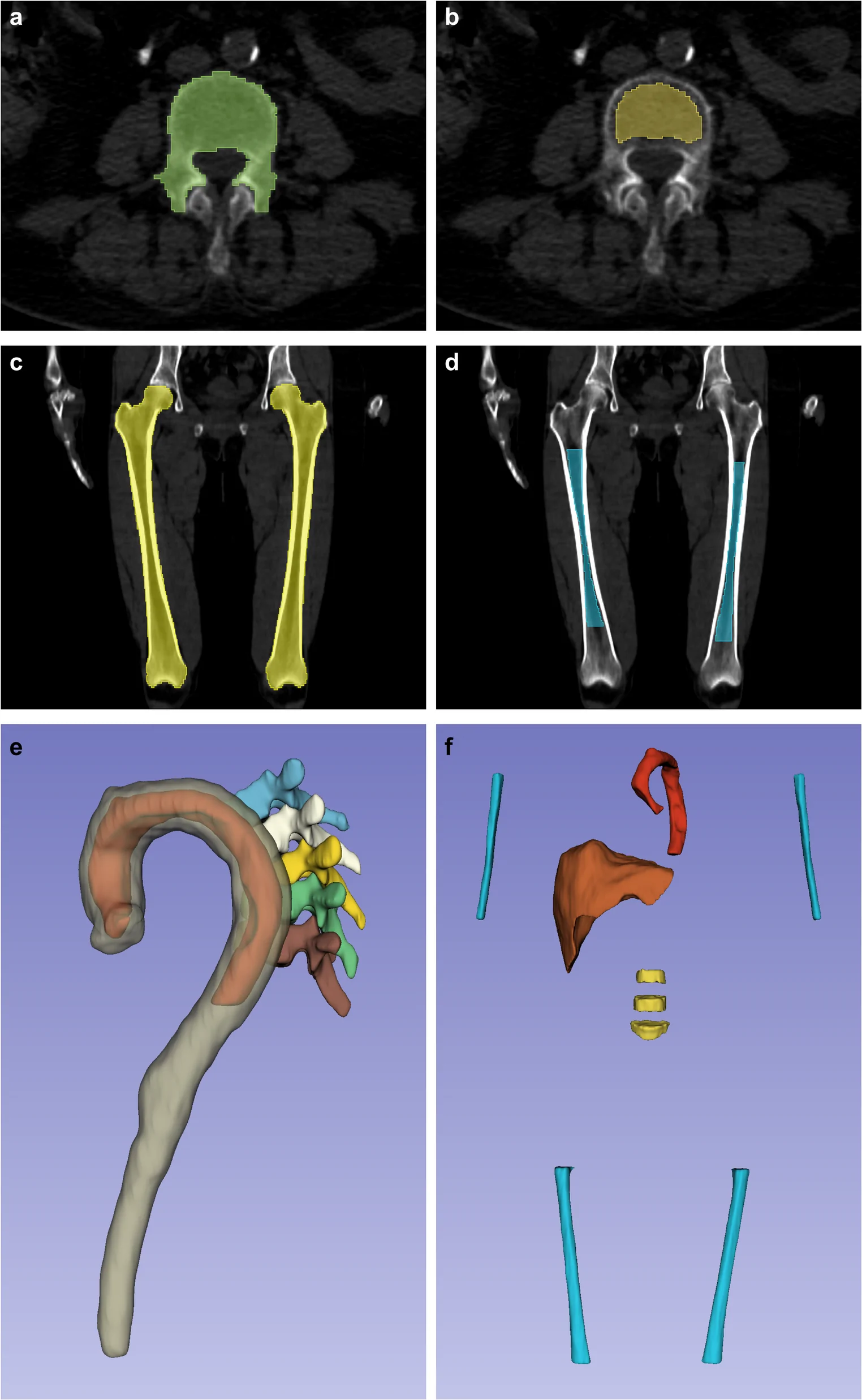
Segmentation refinement for target and reference ROIs. **a**, **b** Vertebrae refinement on axial CT: raw TotalSegmentator mask (**a**, green) and eroded body-only mask (**b**, yellow). **c**, **d** Long bone refinement on coronal CT: femoral segmentation (**c**, yellow) and cropped, eroded diaphyseal mask (**d**, cyan); same procedure applied to humeri. **e**, **f** 3D rendering: e full aorta segmentation (beige) with T4-T8 gating levels and aortic lumen (red); f Final refined masks for DS computation – aortic lumen (red), eroded liver (orange), L3-L5 vertebral bodies (yellow), and femoral/humeral diaphyses (cyan)

Figure 3 illustrates the segmentation refinement pipeline, showing raw and refined masks (a-d) and 3D renderings (e-f). Results from an inter-method agreement analysis between autoDS-PET values and expert visual scoring are reported in Supplementary Material.

### Deauville Scores distribution and prognostic stratification

**Fig. 4:**
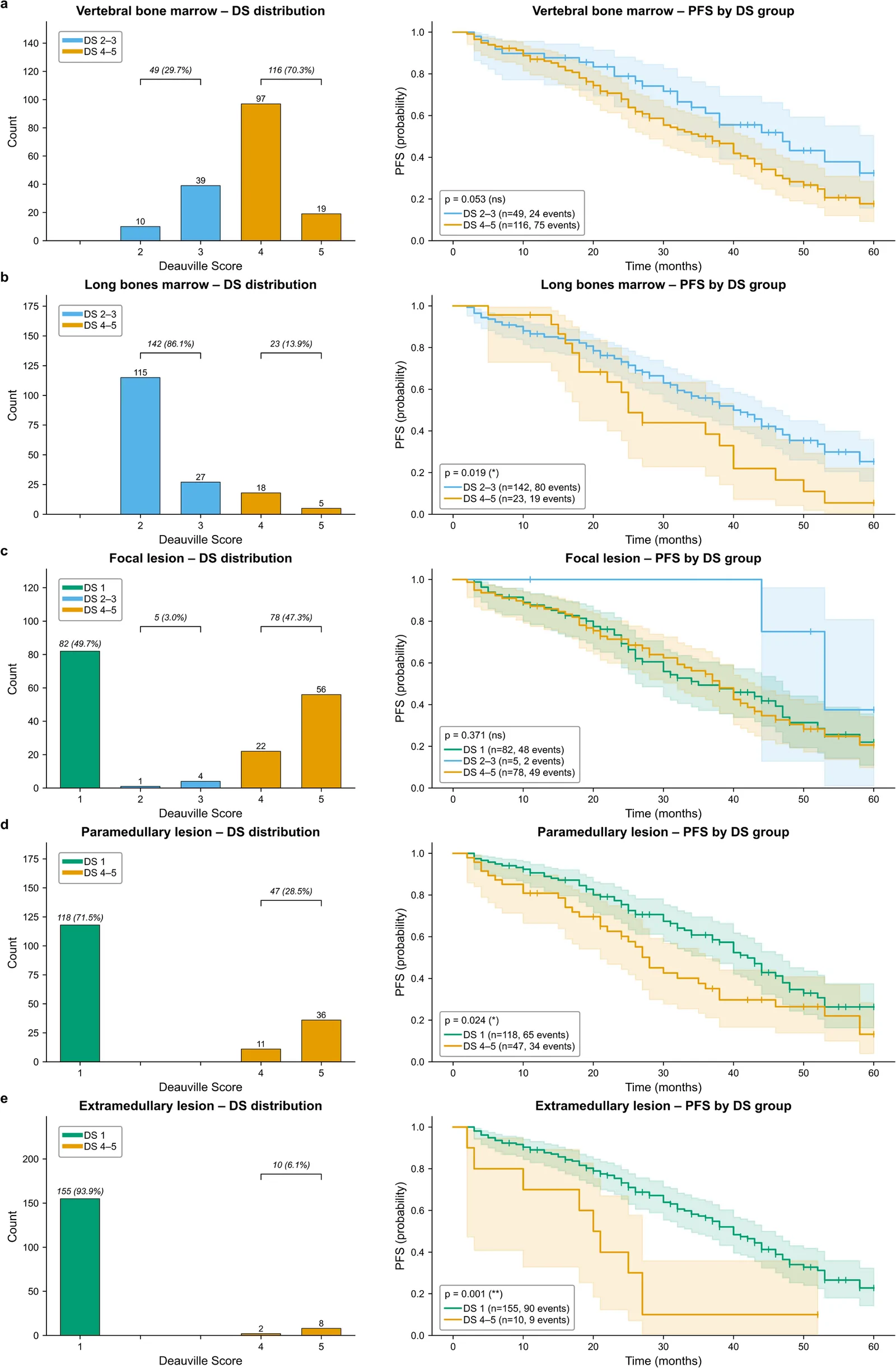
Deauville Score distribution and Kaplan-Meier progression-free survival across five sites. For each site, left panel shows DS distribution grouped into DS 1 (green), DS 2–3 (blue) and DS 4–5 (orange), with counts above bars, and group sizes and percentages in brackets. Right panel shows KM PFS curves stratified by DS group, with 95% pointwise confidence intervals (shaded) and log-rank test p-values; median follow-up time is reported along with its IQR. **a** Vertebral bone marrow; **b** Long bones marrow; **c** Focal lesion; **d** Paramedullary lesion; **e** Extramedullary lesion. Significance notation: * *p* < 0.05; ** *p* < 0.01; ns, not significant

DS distributions and their prognostic stratification varied markedly across the five anatomical sites (Fig. 4). Vertebral BM DS (panel a) was predominantly high (70.3% DS 4–5), with shorter PFS in the high DS group, though not reaching significance (*p* = 0.053). LB marrow (panel b) showed the opposite pattern (86.1% DS 2–3); the smaller DS 4–5 group presented significantly shorter PFS (*p* = 0.019). FL DS (panel c) was balanced between groups but did not discriminate PFS (*p* = 0.371). For PM lesions (panel d), the DS 4–5 group (28.5%) showed significantly shorter PFS (*p* = 0.024) against the DS = 1 group (DS 2–3 group not populated). EM lesions (panel e) were rare (6.1% DS 4–5) but strongly associated with shorter PFS (*p* = 0.001; DS 2–3 group not populated). KM curves with risk tables are reported in Supplementary Material.

### Predictive analysis of PFS

The penalised Cox model for PFS prediction was evaluated in nested CV using 12 covariates and an interaction term: five DS (BM, LB, FL, PM, EM), three CNAs (amp(1q), del(13q), del(17p)), amp(1q)×del(13q) interaction, HB, PLT, age, and sex. Results are reported in Table 4. The model achieved a C-index of 0.710 [95% CI: 0.689–0.732], indicating moderate discriminative ability, and an IBS of 0.175 [95% CI: 0.167–0.183], below the non-informative reference of 0.25 [49], indicating adequate calibration. To assess the added prognostic value of DS, a reduced model including only the seven non-imaging covariates was evaluated, achieving a C-index of 0.672 [95% CI: 0.651–0.693] and an IBS of 0.179 [95% CI: 0.171–0.188]; site-specific DS improved discrimination maintaining comparable calibration.

**Table 4:** Predictive performance of penalised Cox model for PFS. The full model included 12 covariates and an interaction term; the reduced model excluded DS covariates. Discrimination (Uno’s C-index) and calibration (Integrated Brier Score, IBS) evaluated via nested cross-validation (10-fold outer, 5 repetitions). Mean values and 95% confidence intervals from bootstrap resampling (10,000 iterations) of per-fold estimates

Model	C-index [95% CI]	IBS [95% CI]
Full (13 covariates)	0.710 [0.689, 0.732]	0.175 [0.167, 0.183]
Reduced (8 covariates, no DS)	0.672 [0.651, 0.693]	0.179 [0.171, 0.188]

### Associative analysis of prognostic factors

The proportional hazards assumption was satisfied for the multivariable Cox model (*p* = 0.408); scaled Schoenfeld residuals for each covariate are shown in Supplementary Material. All GVIF values were below 1.5 (maximum 1.46, LB DS), confirming the absence of multicollinearity. HR, 95% CI, bootstrap bias of HR estimate, and p-values are reported in Table 5. Age (HR = 1.752, *p* < 0.001), haemoglobin (HR = 0.654, *p* < 0.001), BM DS (HR = 1.238, *p* = 0.021), PM DS (HR = 1.416, *p* = 0.002), and amp(1q) (HR = 1.786, *p* = 0.039) were identified as independent prognostic factors for PFS. Bias obtained by bootstrap simulation confirm the stability of main predictors while highlighting the instability of rarely observed del(17p).

**Table 5:** Associative analysis of prognostic factors for PFS via unpenalised multivariable Cox model fitted on full cohort (*n* = 165, 99 events). For each covariate: hazard ratio (HR), its 95% CIs and *p*-value (single fit), together with bootstrap bias (mean bootstrap coefficient minus single-fit coefficient). Continuous and ordinal variables standardised before fitting. The bias was negligible for all covariates, indicating estimates are robust to sampling variability; the corresponding bootstrap HRs and CI are reported in Supplementary Table 6

Covariate	HR [95% CI]	Bootstrap bias	*p*-value (significance)
Age	1.752 [1.420, 2.160]	+ 0.098	< 0.001 (***)
Sex	1.379 [0.855, 2.223]	+ 0.029	0.187
HB	0.654 [0.519, 0.824]	-0.037	< 0.001 (***)
PLT	0.928 [0.758, 1.136]	+ 0.002	0.469
BM DS	1.238 [1.033, 1.483]	+ 0.022	0.021 (*)
LB DS	0.946 [0.761, 1.176]	+ 0.002	0.618
FL DS	0.993 [0.785, 1.255]	+ 0.007	0.953
PM DS	1.416 [1.138, 1.761]	+ 0.051	0.002 (**)
EM DS	1.154 [1.000, 1.331]	< 0.001	0.050
amp(1q)	1.786 [1.031, 3.096]	+ 0.111	0.039 (*)
del(13q)	1.609 [0.990, 2.616]	+ 0.017	0.055
del(17p)	0.405 [0.103, 1.593]	+ 0.043	0.196
amp(1q)×del(13q)	0.633 [0.297, 1.350]	+ 0.013	0.237

Significance notation: *** *p* < 0.001; ** *p* < 0.01; * *p < 0.05; significant p*-values in bold

## Discussion

This study presents, to our knowledge, the first end-to-end automated pipeline for standardised Deauville Score computation from baseline [^18^F]FDG PET/CT in MM. The pipeline handles the full workflow from DICOM input to DS output, requiring no manual intervention for BM and LB DS; for FL, PM, and EM lesions, semi-automated mode still reduces inter-operator variability by automatically delineating reference regions, and computing and comparing SUV statistics for DS scoring. The reliance on expert-defined lesion segmentations is consistent with current clinical workflows, allowing standardisation of downstream quantification while preserving clinical oversight; future work will target full automation of lesion detection as a functional extension to this process for improved time efficiency, allowing clinicians to review and eventually refine automated outputs rather than segmenting from scratch. The tool ran successfully on all 165 patients across four scanner models and 14 years of clinical practice. Its public availability as an open-source Python package enables independent validation and fosters reproducibility, addressing the recognised lack of standardised quantitative criteria for FDG PET/CT interpretation in MM.

Site-specific univariate KM analysis revealed heterogeneous prognostic patterns. LB DS was significantly associated with shorter PFS (*p* = 0.019), despite 86.1% of patients being classified as DS 2–3. In adults, long bone diaphyses are predominantly occupied by fatty (yellow) marrow, a non-haematopoietic compartment; elevated FDG uptake at these sites may reflect disease involvement in appendicular skeleton. Recently, Sachpekidis et al. [50] demonstrated that patients with DS ≥ 4 in bone marrow exhibit significantly higher appendicular CT values correlated with histopathologically-confirmed plasma cell infiltration; Jamet et al. [51] recognised extension of diffuse uptake to peripheral skeleton as a marker of more extensive disease. Complementary findings by Schinke et al. [52], although focused on focal rather than diffuse disease, showed that lesion location in long bones is independently associated with adverse outcomes. This supports extending metabolic assessment beyond the axial skeleton – a compartment not currently scored in IMPeTUs criteria. EM DS was strongly associated with shorter PFS (*p* = 0.001), consistent with the aggressive biology of EM disease [53]; nonetheless, its occurrence in the dataset is limited (6.1% of patients), warranting further validation in cohorts with a larger occurrence of rare events. PM DS also reached significance (*p* = 0.024). FL DS did not discriminate PFS outcomes. The IMPeTUs framework separates FL assessment into two parameters: DS of the hottest lesion (intensity, computed by autoDS-PET) and FL count (burden). Recent evidence indicates that FL count, rather than intensity, is the primary driver of prognosis [54, 55], suggesting that metabolic intensity of the single hottest lesion does not capture the full prognostic information carried by overall FL burden. Integration of automated FL detection and counting is identified as a priority for future development towards a more complete disease assessment.

The multivariable associative analysis identified age, haemoglobin, BM DS, PM DS, and amp(1q) as independent prognostic factors for PFS, reinforcing the complementary value of clinical, laboratory, CNA, and imaging variables. The protective effect of higher haemoglobin (HR = 0.654) aligns with the well-established adverse prognostic role of anaemia in MM reflecting greater BM infiltration and compromised haematopoiesis. Among imaging-derived variables, BM DS and PM DS reached significance in the multivariable model (HR = 1.238, *p* = 0.021; HR = 1.416, *p* = 0.002), highlighting the prognostic relevance of diffuse disease in BM and disease extending beyond medullary cavity into adjacent soft tissues. Among genomic variables, amp(1q), carried by 60 patients, was significantly associated with shorter PFS in the multivariable model (HR = 1.786, *p* = 0.039), consistent with its established adverse prognostic role in MM [24]. The amp(1q)×del(13q) interaction term did not reach significance (HR = 0.633, *p* = 0.237), possibly because only 34 patients carried both alterations, limiting statistical power to detect such an interaction. The counterintuitive protective HR for del(17p) (HR = 0.405 [0.103, 1.593], *p* = 0.196) is not statistically significant, and should not be interpreted as evidence of a protective effect; it likely reflects sparse-data bias, as only 11 patients (7%) carried this alteration, yielding insufficient statistical power to reliably estimate its contribution. Sensitivity analysis excluding rare events confirmed that unstable estimates do not affect the main findings (Supplementary Material). Larger cohorts are needed to reliably estimate the prognostic impact of rare events.

In predictive analysis, the penalised Cox model achieved a C-index of 0.710, reflecting moderate discrimination. A reduced model excluding the five DS variables yielded a lower C-index (0.672), confirming that site-specific metabolic imaging features add prognostic value beyond CNA and clinical variables. This improvement reflects the combined prognostic contribution of all DS features, including those not individually significant in the associative analysis [56]. The low-dimensional feature set (12 covariates) was chosen to balance interpretability with statistical robustness for the available sample size. Analogous OS analyses yielded broadly consistent results (C-index 0.699 [0.655, 0.739]; Supplementary Material), supporting robustness across endpoints. By preserving direct alignment with established clinical reporting criteria, this approach prioritises standardisation and interpretability, offering a reproducible alternative to high-dimensional radiomics.

This study has some limitations. First, its retrospective monocentric design limits generalisability; external validation on independent cohorts remains essential for clinical translation and a priority for future work. Second, the long enrolment period (2007–2021) encompasses changes in frontline treatment regimens; treatment heterogeneity may confound PFS outcomes (sensitivity analyses adjusting for treatment regimen confirmed main findings’ stability, Supplementary Material). Third, the sample size, while adequate for the number of covariates, limited statistical power for rare events such as del(17p) (*n* = 11) and EM disease (*n* = 10). Fourth, genomic characterisation was limited to three CNAs and did not include the full cytogenetic and molecular profile (t(4;14) and t(14;16), notably); the inclusion of a more comprehensive genomic profile could refine the predictive model and better capture the biological heterogeneity of the disease. Future work will prioritise external validation on independent cohorts and exploration of the framework’s potential integration into clinical workflows to support patients’ stratification at diagnosis. The autoDS-PET pipeline is released as a research tool.

In conclusion, this work demonstrates feasibility and clinical relevance of automated DS computation from baseline FDG PET/CT in NDMM. The integration of site-specific DS with CNA and clinical variables enables a multimodal framework for PFS prediction, and the publicly available pipeline offers a reproducible tool that may support standardisation efforts in PET/CT interpretation for MM.

## Supplementary Information

Below is the link to the electronic supplementary material.

Supplementary Material 1

## Data Availability

The datasets generated and analysed during the current study may be made available from the corresponding author upon reasonable request and subject to institutional approval.

## Code availability

The pipeline for automatic and semi-automatic DS computation (*autoDS-PET*) is publicly available as an installable Python package at https://github.com/Sara-Peluso/autoDS-PET with customisable settings. The GitHub repository includes source code and usage instructions.
