# Supplementary material for: Automated Deauville Score computation from baseline [¹⁸F]FDG PET/CT predicts progression-free survival in multiple myeloma: a radiogenomic framework
Source: Eur J Nucl Med Mol Imaging. 2026 Jul 30;53(12):7336–49. doi: 10.1007/s00259-026-08103-x (PMC13633357; doi:10.1007/s00259-026-08103-x)
Supplement: Supplementary file 1 [file 259_2026_8103_MOESM1_ESM.pdf]

Supplementary Material for:
Automated Deauville Score computation from baseline [^{18}F]FDG PET/CT predicts progression-free survival in multiple myeloma: a radiogenomic framework

Sara Peluso[1,2,†,*], Stefano Polizzi[3,†], Lisa Pagnini[1,4], Martina Tarozzi[1,2], Ettore Rocchi[1,2], Valentino Dragonetti[5], Vincenza Solli[1,6], Daniele Dall'Olio[1], Marco Talarico[1,6], Carolina Terragna[6], Claudia Sala[1,2], Elena Zamagni[1,6], Stefano Fanti[1,4], Cristina Nanni[4,‡] and Gastone Castellani[1,2,‡]

[1]Department of Medical and Surgical Sciences, University of Bologna, 40138 Bologna (Italy)
[2]IRCCS Azienda Ospedaliero Universitaria di Bologna, 40138 Bologna (Italy)
[3]IRCCS Istituto delle Scienze Neurologiche di Bologna, Data Science and Bioinformatics Laboratory, 40139 Bologna, Italy
[4]Nuclear Medicine, IRCCS Azienda Ospedaliero-Universitaria di Bologna, Bologna (Italy)
[5]Division of Nuclear Medicine, IEO European Institute of Oncology IRCCS, Milan, Italy
[6]IRCCS Azienda Ospedaliero-Universitaria di Bologna, Istituto di Ematologia "Seràgnoli", Bologna (Italy)

† These authors contributed equally to this work and share first authorship.
‡ These authors contributed equally to this work and share last authorship.

* Corresponding author: sara.peluso5@unibo.it

Contents

S1 Distribution and Kaplan-Meier analysis of non-imaging covariates **3**

S2 Overall survival analysis **6**

S3 Proportional hazards diagnostics **7**

S4 Sensitivity analysis on translocations **8**

S5 Bootstrap on HR estimates **9**

S6 Kaplan-Meier curves with risk tables **11**

S7 Follow-up duration for each DS subgroup **12**

S8 Inter-method agreement analysis on Deauville Score **13**

S9 Sensitivity analyses on treatment **15**

S9.1 Anti-CD38-containing model 15
S9.2 IMiD-containing model 15
S9.3 PI-containing model 16

S10 Comparison with R-ISS staging **18**

S11 Sensitivity analysis on rare events **19**

S1 Distribution and Kaplan-Meier analysis of non-imaging covariates

Kaplan-Meier (KM) progression-free survival (PFS) curves were estimated for the seven non-imaging covariates included in the radiogenomic feature set. Continuous variables were dichotomised at HB = 10 g/dL (IMWG CRAB anaemia criterion), PLT = 150 ×109/L (thrombocytopenia threshold), and age = 65 years; sex and the three copy number alterations (CNAs) were stratified by native categories. Groups were compared using the log-rank test. In all panels except sex, the orange curve denotes the group with the a priori adverse prognostic profile (HB <10 g/dL, PLT <150 ×109/L, age ≥65 years, CNA carriers) and blue denotes the reference group. Results are presented in three figures grouped by biological category: demographic variables (Supplementary Fig. 1), blood testing variables (Supplementary Fig. 2), and genomic variables (Supplementary Fig. 3).

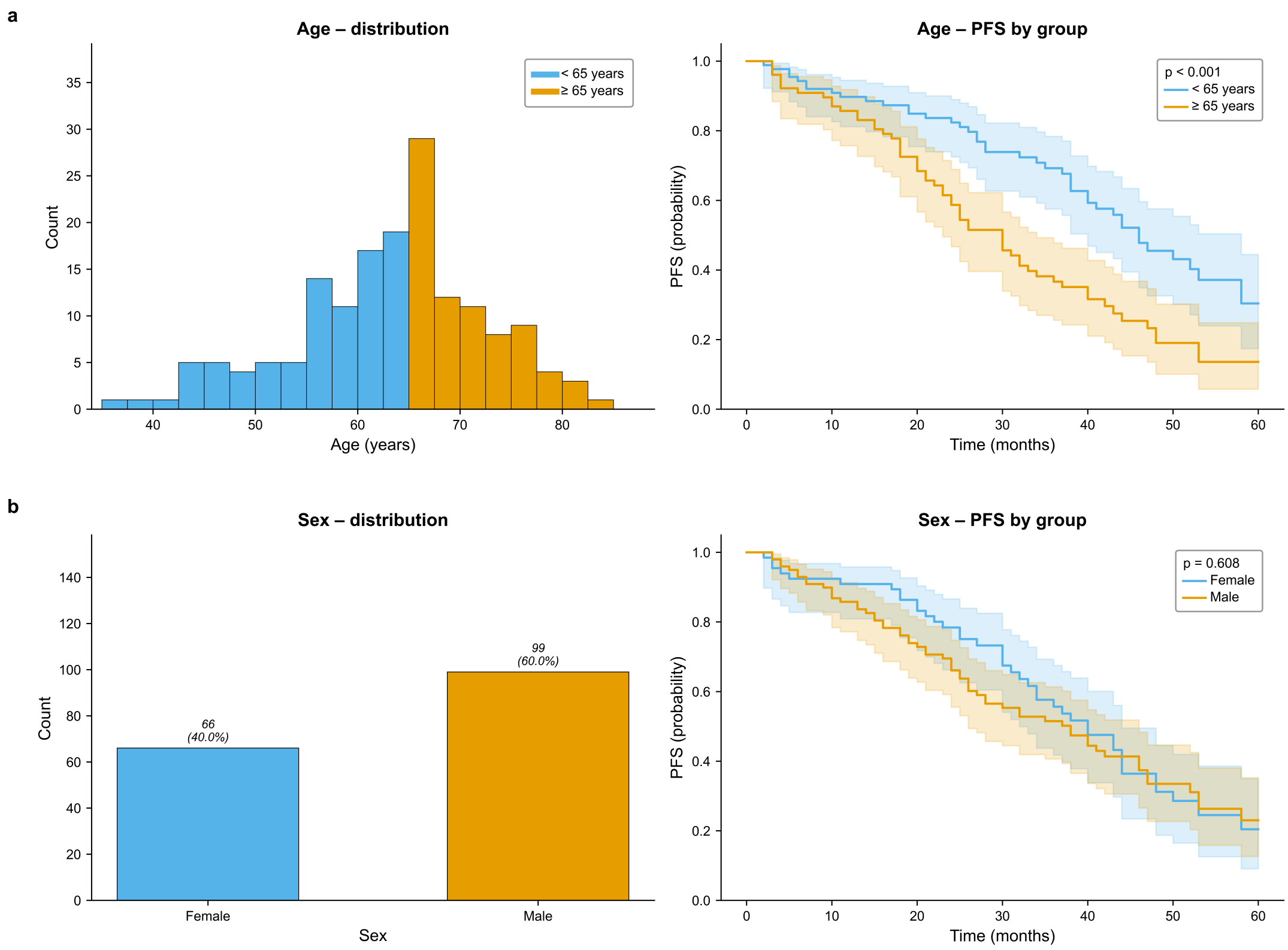

Supplementary Figure 1: Kaplan-Meier PFS curves by age (a) and biological sex (b) in the study cohort (n = 165). Age was dichotomised at ≥65 years (orange) vs <65 years (blue); for sex, the female/male colour pairing is descriptive only and does not encode a prognostic direction. Log-rank p-values are reported in each legend. Patients aged ≥65 years had significantly shorter PFS ($p < 0.001$); no difference was observed by sex

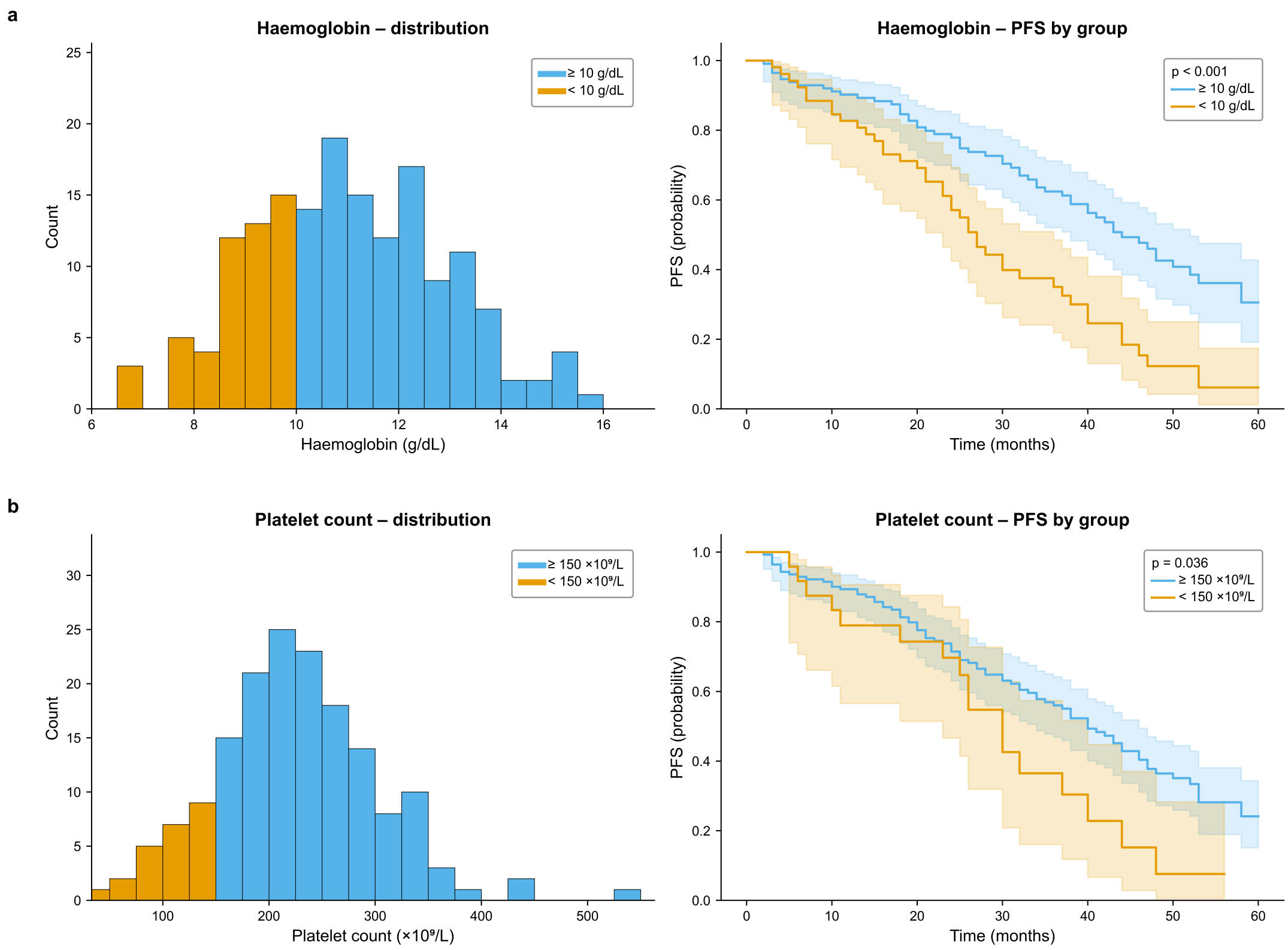

Supplementary Figure 2: Kaplan-Meier PFS curves by haemoglobin (a) and platelet count (b). HB <10 g/dL and PLT <150 ×109/L are shown in orange; reference groups (≥10 g/dL, ≥150 ×109/L) in blue. Log-rank p-values are reported in each legend. HB <10 g/dL was associated with significantly shorter PFS ($p < 0.001$); PLT <150 ×109/L was associated with significantly shorter PFS ($p = 0.036$)

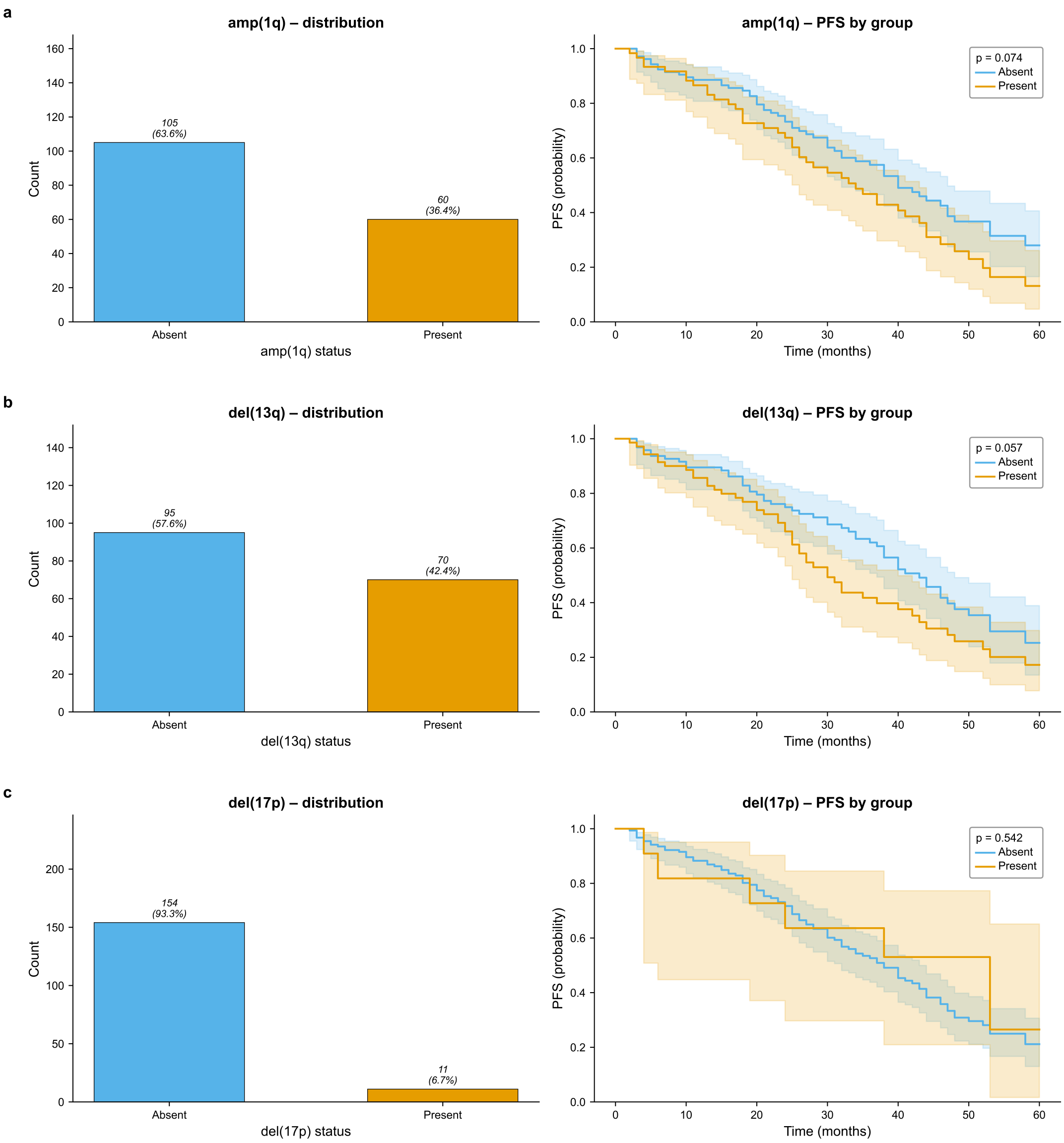

Supplementary Figure 3: Kaplan-Meier PFS curves by amp(1q) (a), del(13q) (b), and del(17p) (c), stratified by carrier status (Absent, blue; Present, orange). Log-rank p-values are reported in each legend. None reached significance. del(17p) curves should be interpreted with caution given the small number of carriers (n = 11, 7%), consistent with the sparse-data instability noted in the main text for the multivariable Cox estimate

S2 Overall survival analysis

To assess the robustness of the prognostic framework across endpoints, the predictive and associative analyses described in the main text were replicated using overall survival (OS) as the endpoint. 48 OS events were observed over a median follow-up of 51 months. Median follow-up was estimated using the reverse KM method. Supplementary Table 1 reports the predictive performance of the full and ablation models for OS. Supplementary Table 2 reports the associative analysis results. Overall, findings were broadly consistent with the PFS analysis.

Model	C-index [95% CI]	IBS [95% CI]
Full (12 covariates)	0.699 [0.655, 0.739]	0.193 [0.172, 0.216]
Reduced (7 covariates, no DS)	0.622 [0.578, 0.665]	0.213 [0.190, 0.237]

Supplementary Table 1: Predictive performance of the penalised Cox model for OS. The full model included 12 covariates (five Deauville Scores, DS; three CNAs; haemoglobin, platelet count, age, sex); the reduced model excluded the five DS variables. Model discrimination was assessed using Uno's C-index and model calibration using the integrated Brier score (IBS), both evaluated via nested cross-validation (10-fold outer, 5 repetitions). Mean values and 95% confidence intervals were obtained by bootstrap resampling (10,000 iterations) of the per-fold estimates

Covariate	HR [95% CI]	p-value
Age	1.855 [1.314, 2.619]	$<$ **0.001** (***)
Sex	1.495 [0.796, 2.809]	0.212
HB	0.919 [0.600, 1.406]	0.696
PLT	0.656 [0.385, 1.117]	0.120
BM DS	1.223 [0.908, 1.646]	0.185
LB DS	0.832 [0.598, 1.157]	0.275
FL DS	0.974 [0.683, 1.390]	0.884
PM DS	1.874 [1.400, 2.508]	$<$ **0.001** (***)
EM DS	1.265 [0.997, 1.604]	0.053
amp(1q)	2.569 [1.066, 6.188]	**0.035** (*)
del(13q)	1.867 [0.892, 3.906]	0.098
del(17p)	0.935 [0.306, 2.857]	0.906
amp(1q)×del(13q)	0.372 [0.122, 1.129]	0.081

Supplementary Table 2: Associative analysis of prognostic factors for PFS. An unpenalised multivariable Cox proportional hazards model was fitted on the full cohort ($n = 165$, 99 events). Hazard ratios (HR) with 95% confidence intervals and Wald test p-values are reported for each covariate. Continuous and ordinal variables were standardised prior to fitting. Significance notation: *** $p < 0.001$; ** $p < 0.01$; * $p < 0.05$. Significant p-values are in bold

S3 Proportional hazards diagnostics

The proportional hazards assumption of the multivariable Cox model was assessed using a global Grambsch-Therneau test on scaled Schoenfeld residuals, yielding $\chi^2 = 14.281$ with df = 13 (p = 0.354), indicating no evidence of violation. Supplementary Fig. 4 shows scaled Schoenfeld residuals versus event time for each covariate, with a locally weighted scatterplot smoothing (LOWESS) curve overlaid to visualise departures from the zero-slope null expectation.

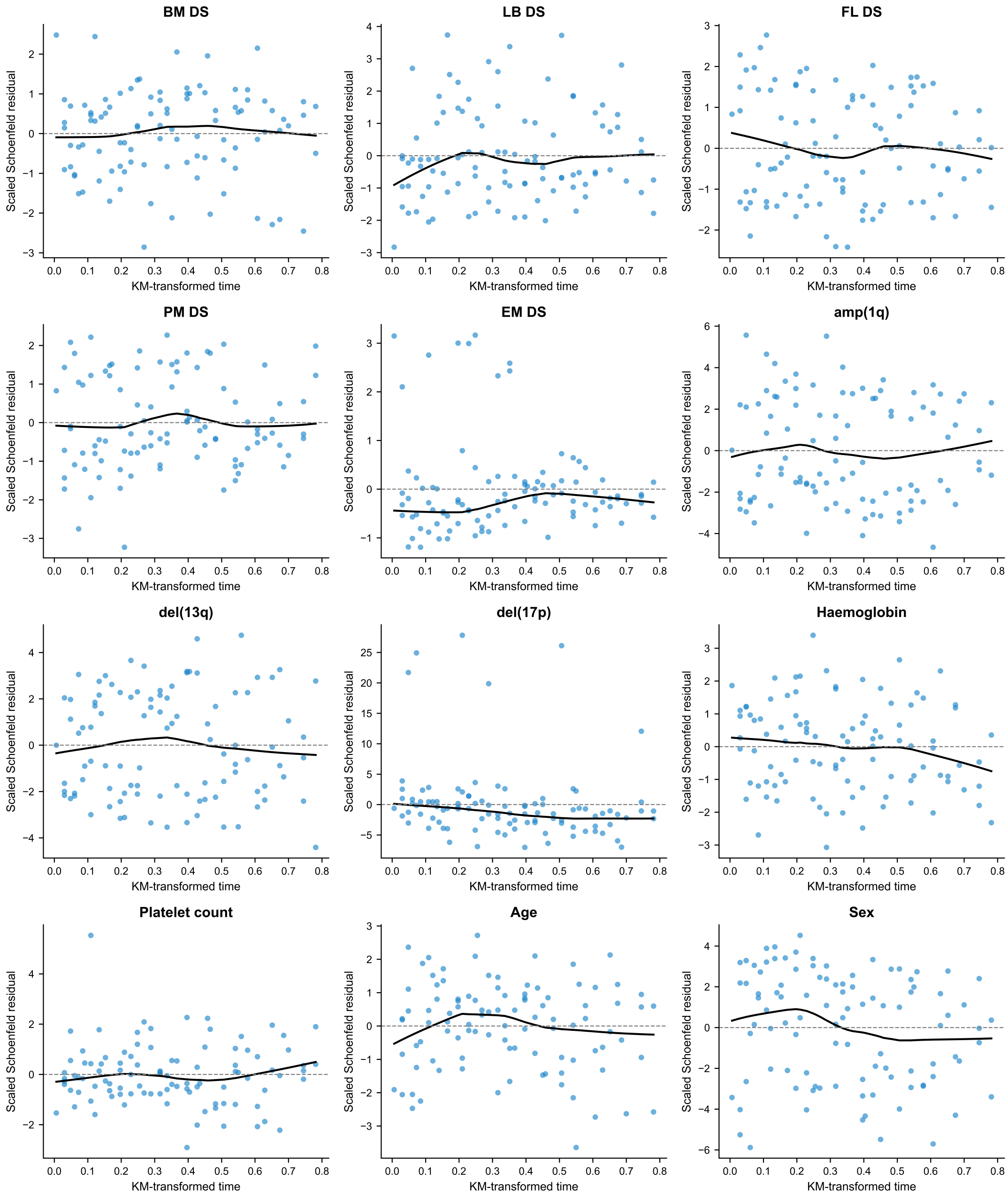

Supplementary Figure 4: Scaled Schoenfeld residuals versus Kaplan-Meier transformed event time for the 12 covariates of the multivariable Cox model with LOWESS curve. Residuals are centred around zero across the follow-up window. BM: bone marrow; LB: long bones; FL: focal lesions; PM: paramedullary; EM: extramedullary; DS: Deauville Score

S4 Sensitivity analysis on translocations

Considering the inclusion of IgH translocations t(4;14) and t(14;16), the dataset was narrowed to 147 patients, presenting 86 PFS events. Given that del(17p) and t(14;16) are both established high-risk markers whose co-occurrence defines "double-hit" myeloma, a subgroup associated with markedly adverse outcomes [1,2], a t(14;16)×del(17p) interaction term was included to account for potential confounding between these rare, biologically related alterations. The predictive model yielded a C-index of 0.712 [0.691, 0.730] and an IBS of 0.176 [0.167, 0.185]. The associative analysis is reported in Supplementary Table 3.

Covariate	HR [95% CI]	|Variation (%)|	p-value
Age	1.719 [1.298, 2.276]	0.88	< **0.001** (***)
Sex	1.331 [0.791, 2.241]	7.13	0.282
HB	0.555 [0.421, 0.731]	14.58	< **0.001** (***)
PLT	0.907 [0.717, 1.146]	0.20	0.413
BM DS	1.231 [1.003, 1.511]	2.43	**0.047** (*)
LB DS	0.968 [0.740, 1.266]	7.45	0.812
FL DS	1.042 [0.798, 1.360]	0.14	0.765
PM DS	1.541 [1.211, 1.961]	3.33	< **0.001** (***)
EM DS	1.272 [0.990, 1.634]	6.74	0.060
amp(1q)	1.573 [0.859, 2.880]	2.80	0.142
del(13q)	1.757 [1.037, 2.976]	8.66	**0.036** (*)
del(17p)	1.002 [0.333, 3.017]	194.54	0.997
amp(1q)×del(13q)	0.855 [0.363, 2.012]	25.37	0.719
t(4;14)	0.764 [0.338, 1.725]	—	0.517
t(14;16)	0.241 [0.039, 1.481]	—	0.125
t(14;16)×del(17p)	0.178 [0.023, 1.369]	—	0.097

Supplementary Table 3: Associative analysis of prognostic factors for PFS with the addition of two high-risk IgH translocations: t(4;14) and t(14;16), and t(14;16)×del(17p) interaction term. An unpenalised multivariable Cox proportional hazards model was fitted on the restricted cohort (n = 147, 86 events). Hazard ratios (HR) with 95% confidence intervals and p-values are reported for each covariate. Continuous and ordinal variables were standardised prior to fitting. Significance notation: *** $p < 0.001$; ** $p < 0.01$; * $p < 0.05$. Significant p-values are in bold

S5 Bootstrap on HR estimates

The stability of the multivariable Cox model was assessed with a non-parametric bootstrap (n = 165, B = 2000 resamples with replacement), refitting the full model on each resample. Median bootstrap HRs were close to the single-fit estimates with negligible bias, confirming that the results are robust to sampling variability. The full sampling distribution of each HR across the 2000 bootstrap resamples was examined. For every covariate in the multivariable Cox model (including the amp(1q)×del(13q) interaction), the distribution of bootstrap HRs is shown in Supplementary Fig. 5. This visualises the precision and shape (e.g. skewness) of each estimate and whether its 95% bootstrap confidence interval excludes the null value of 1.

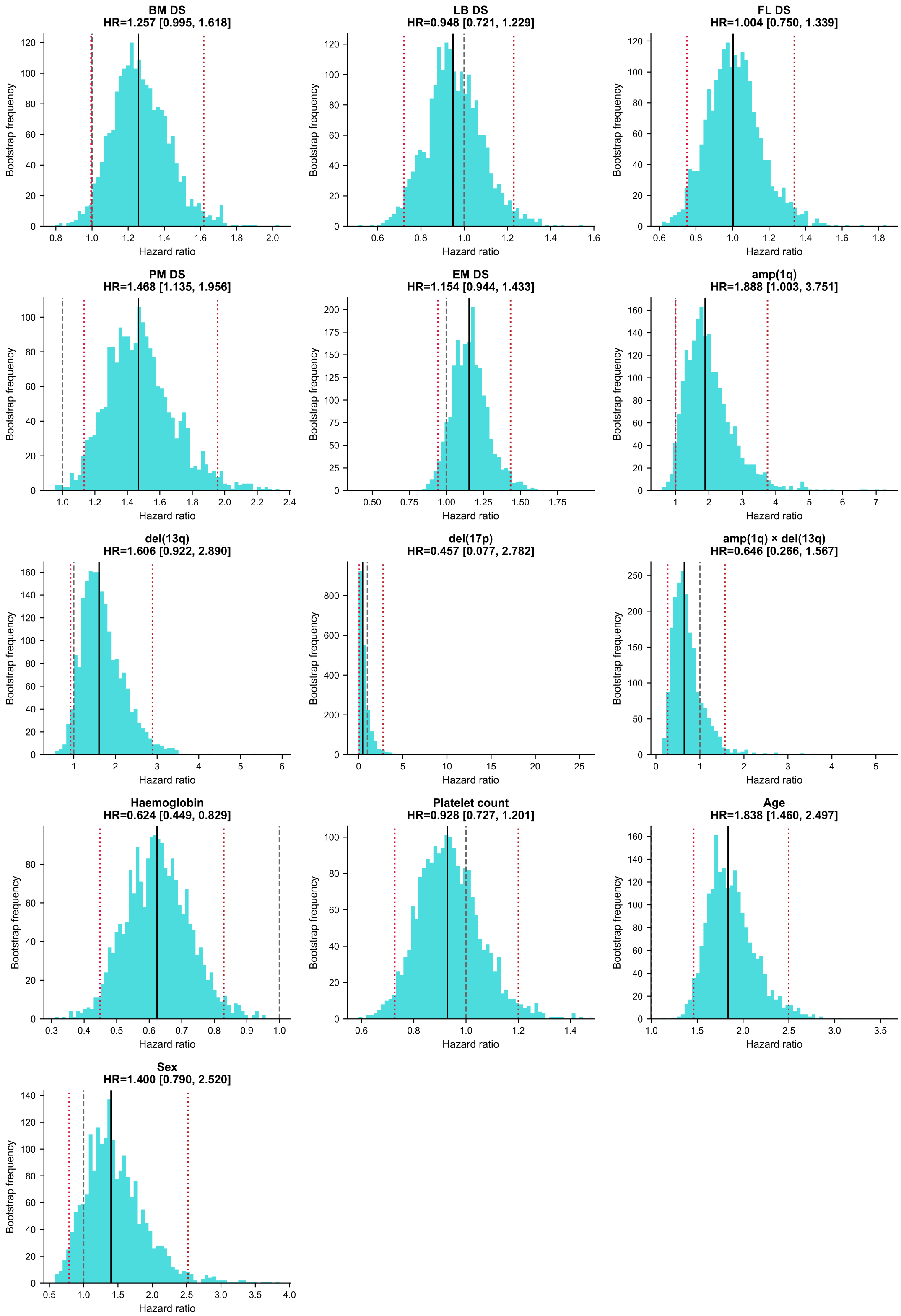

Supplementary Figure 5: Bootstrap distributions of hazard ratios for each covariate of the multivariable Cox model. Each panel shows the histogram of HR obtained from 2000 bootstrap resamples of patients (n = 165) with replacement. In each panel, the solid black line marks the median bootstrap HR, the dotted red lines the 2.5th and 97.5th percentiles (95% bootstrap percentile CI), and the dashed grey line the null value (HR = 1); the median HR and its 95% CI are also reported above each panel. Covariates whose CI lies entirely to one side of HR = 1 indicate a stable association across resamples

S6 Kaplan-Meier curves with risk tables

Kaplan-Meier curves with number-at-risk tables and cumulative event counts at regular time intervals are displayed in Supplementary Fig. 6.

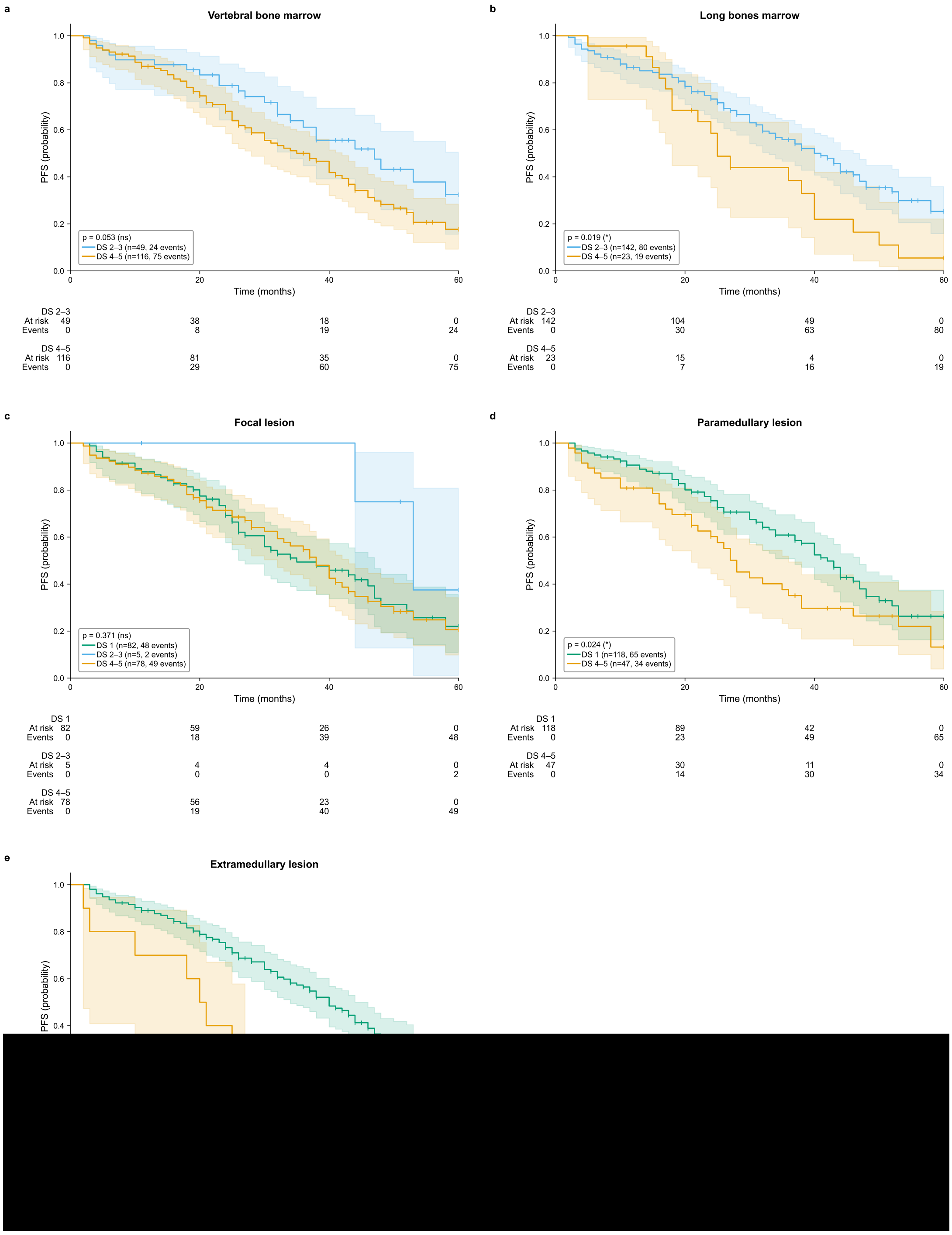

Supplementary Figure 6: Kaplan-Meier progression-free survival (PFS) curves by DS group across five anatomical sites, with number-at-risk and cumulative event counts reported at regular time intervals. Group sizes, number of events, and log-rank p-values are as reported in Fig. 4 of the main text. **a** Vertebral bone marrow (DS 2-3 vs. DS 4-5); **b** Long bones marrow (DS 2-3 vs. DS 4-5); **c** Focal lesion (DS 1 vs. DS 2-3 vs. DS 4-5); **d** Paramedullary lesion (DS 1 vs. DS 4-5); **e** Extramedullary lesion (DS 1 vs. DS 4-5). Censoring events are indicated by vertical dashes; shaded areas represent 95% pointwise confidence intervals

S7 Follow-up duration for each DS subgroup

Referring to the DS subgroups presented in Fig. 4 of main text, median follow-up time for each subgroup is reported in Supplementary Table 4, along with the interquartile range (IQR).

**Site**	**Group**	**n**	**Median follow-up [IQR] (months)**
Vertebral bone marrow	DS 2-3	49	46 [34, 60]
	DS 4-5	116	51 [37, 60]
Long bones marrow	DS 2-3	142	50 [37, 60]
	DS 4-5	23	60 [60, 60]
Focal lesion	DS 1	82	45 [32, 60]
	DS 2-3	5	60 [51, 60]
	DS 4-5	78	51 [38, 60]
Paramedullary lesion	DS 1	118	50 [36, 60]
	DS 2-3	0	—
	DS 4-5	47	52 [42, 60]
Extramedullary lesion	DS 1	155	50 [37, 60]
	DS 2-3	0	—
	DS 4-5	10	52 [52, 52]

Supplementary Table 4: Follow-up time duration for each DS subgroup used in Kaplan-Meier curves; median and IQR reported

S8 Inter-method agreement analysis on Deauville Score

To assess the reliability of automated DS computation, inter-method agreement between autoDS-PET and expert visual scoring was evaluated on the full cohort (n = 165). Expert DS were assigned by an experienced nuclear medicine physician following the IMPeTUs criteria. Agreement was quantified using percentage of exact agreement, Cohen's kappa, and Krippendorff's alpha. Two levels of granularity were assessed: exact agreement on the 5-point DS scale (Supplementary Table 5), and agreement on clinically relevant groups reflecting the DS subgroupings used for Kaplan-Meier curves (Supplementary Table 6). For BM and LB, where all patients have the anatomical compartment, the grouping was DS 2-3 vs. DS 4-5; for FL, PM, and EM, where DS 1 indicates absence of detectable disease, the grouping was DS 2-3 vs. DS 4-5 too, because if no lesion segmentation is provided the autoDS-PET computation is not computed for that compartment. Supplementary Table 5 reports exact agreement on the 5-point DS scale. The lower exact agreement for BM and LB DS is consistent with the known difficulty of discriminating DS 2 and DS 3 in diffuse marrow assessment, where bone marrow uptake near the mediastinal reference threshold is visually ambiguous – a challenge also reported among expert readers in the IMPeTUs reproducibility study (Krippendorff's alpha = 0.23-0.53 for BM DS, from Nanni et al. [3]). Supplementary Table 6 reports agreement after grouping DS into clinically relevant categories used in the survival analysis (DS 2-3 vs DS 4-5). Grouped agreement improved substantially for all applicable sites. PM and EM are not reported in Supplementary Table 6 because all patients with detectable lesions at these sites had DS 4-5 in both automated and expert scoring, leaving no DS 2-3 subgroup for comparison (blank in the table); this itself reflects high concordance for these sites.

**DS by site**	**Matching cases**	**Agreement (%)**	**Cohen's kappa**	**Krippendorff's alpha**
BM DS	105/165	63.64%	0.48	0.70
LB DS	101/165	61.62%	0.44	0.59
FL DS	62/83	74.70%	0.54	0.70
PM DS	33/47	70.21%	0.42	0.63
EM DS	9/10	90.00%	0.95	0.74

Supplementary Table 5: Inter-method agreement results between automated (autoDS-PET) and expert-assigned Deauville Scores by exact value (5-point scale). Agreement assessed on the full cohort (n = 165) for each anatomical site

**DS by site**	**Grouping**	**Matching cases**	**Agreement (%)**	**Cohen's kappa**	**Krippendorff's alpha**
BM DS	2-3 vs. 4-5	140/165	84.85%	0.70	0.70
LB DS	2-3 vs. 4-5	135/165	81.82%	0.61	0.60
FL DS	2-3, 4-5	77/83	92.77%	0.54	0.53
PM DS	2-3, 4-5	—	—	—	—
EM DS	2-3, 4-5	—	—	—	—

Supplementary Table 6: Inter-method agreement between automated (autoDS-PET) and expert-assigned Deauville Scores by clinically relevant groupings: DS 2-3 vs. DS 4-5 for BM and LB (where all patients have the anatomical compartment); DS 2-3 vs. DS 4-5 for FL DS (among patients with detectable lesions). PM and EM are not reported as all patients with detectable lesions were assigned DS 4-5 by both methods, precluding subgroup comparison

S9 Sensitivity analyses on treatment

The enrolment period (2007-2021) spans significant changes in frontline therapy for MM, most notably the introduction of anti-CD38 monoclonal antibodies into induction regimens. To assess whether treatment heterogeneity confounds the primary findings, the predictive and associative models were refitted with the addition of a binary treatment covariate. Three separate analyses were performed, each capturing a different dimension of treatment variation: (i) IMiD-containing regimen (n=118 yes, n=47 no); (ii) PI-containing regimen (n=152 yes, n=13 no); (iii) anti-CD38-containing regimen (n=20 yes, n=145 no). The |Variation (%)| column reports the absolute percentage change in HR relative to the primary model without treatment adjustment, computed as $|HR\_adjusted - HR\_primary| / HR\_primary \times 100$. Predictive performance (C-index, IBS) for each model is reported alongside the associative results.

S9.1 Anti-CD38-containing model

Out of 165 patients, 20 underwent a therapy containing anti-CD38, 145 did not. The predictive analysis yielded: C-index 0.707 [0.685, 0.729], IBS 0.175 [0.167, 0.183]. Associative analysis results are reported in Supplementary Table 7.

Covariate	HR [95% CI]	|Variation (%)|	p-value
Age	1.768 [1.426, 2.191]	0.92	< **0.001** (***)
Sex	1.364 [0.850, 2.187]	1.11	0.198
HB	0.654 [0.518, 0.824]	0.03	< **0.001** (***)
PLT	0.930 [0.759, 1.140]	0.26	0.487
BM DS	1.233 [1.025, 1.484]	0.36	**0.026** (*)
LB DS	0.950 [0.762, 1.184]	0.36	0.646
FL DS	0.985 [0.777, 1.250]	0.78	0.903
PM DS	1.414 [1.138, 1.757]	0.14	**0.013** (*)
EM DS	1.145 [0.984, 1.332]	0.80	**0.002** (**)
amp(1q)	1.782 [1.029, 3.085]	0.25	**0.039** (*)
del(13q)	1.580 [0.971, 2.570]	1.82	0.066
del(17p)	0.393 [0.099, 1.554]	3.10	0.183
amp(1q)×del(13q)	0.656 [0.265, 1.244]	3.61	0.277
Anti-CD38-containing	0.753 [0.307, 1.402]	—	0.492

Supplementary Table 7: Associative analysis of prognostic factors for PFS adding anti-CD38 treatment binary covariate. An unpenalised multivariable Cox proportional hazards model was fitted on the full cohort (n = 165, 99 events). Hazard ratios (HR) with 95% confidence intervals and Wald test p-values are reported for each covariate. Continuous and ordinal variables were standardised prior to fitting. Significance notation: *** $p < 0.001$; ** $p < 0.01$; * $p < 0.05$. Significant p-values are in bold

S9.2 IMiD-containing model

Out of 165 patients, 118 underwent a therapy containing IMiD, 48 did not. The predictive analysis yielded: C-index 0.704 [0.681, 0.727], IBS 0.174 [0.166, 0.182]. Associative analysis results are reported in Supplementary Table 8.

Covariate	HR [95% CI]	|Variation (%)|	p-value
Age	1.743 [1.413, 2.150]	0.48	< **0.001** (***)
Sex	1.403 [0.882, 2.230]	1.71	0.153
HB	0.642 [0.511, 0.806]	1.86	< **0.001** (***)
PLT	0.948 [0.779, 1.154]	2.16	0.594
BM DS	1.290 [1.069, 1.556]	4.18	**0.008** (**)
LB DS	0.917 [0.735, 1.143]	3.13	0.440
FL DS	1.010 [0.796, 1.282]	1.73	0.934
PM DS	1.414 [1.132, 1.766]	0.15	**0.002** (**)
EM DS	1.145 [0.981, 1.336]	0.78	0.086
amp(1q)	1.879 [1.101, 3.206]	1.88	**0.021** (*)
del(13q)	1.688 [1.039, 2.742]	1.69	**0.035** (*)
del(17p)	0.421 [0.111, 1.586]	0.42	0.201
amp(1q)×del(13q)	0.585 [0.273, 1.257]	0.59	0.169
IMiD-containing	0.651 [0.432, 0.982]	—	**0.041** (*)

Supplementary Table 8: Associative analysis of prognostic factors for PFS adding IMiD-containing treatment binary covariate. An unpenalised multivariable Cox proportional hazards model was fitted on the full cohort (n = 165, 99 events). Hazard ratios (HR) with 95% confidence intervals and Wald test p-values are reported for each covariate. Continuous and ordinal variables were standardised prior to fitting. Significance notation: *** $p < 0.001$; ** $p < 0.01$; * $p < 0.05$. Significant p-values are in bold

S9.3 PI-containing model

Out of 165 patients, 153 underwent a therapy containing PI, 13 did not. The predictive analysis yielded: C-index 0.710 [0.689, 0.732], IBS 0.175 [0.167, 0.183]. Associative analysis results are reported in Supplementary Table 9.

Covariate	HR [95% CI]	|Variation (%)|	p-value
Age	1.685 [1.357, 2.093]	3.78	< **0.001** (***)
Sex	1.347 [0.831, 2.183]	2.33	0.227
HB	0.642 [0.508, 0.812]	1.81	< **0.001** (***)
PLT	0.934 [0.761, 1.146]	0.62	0.513
BM DS	1.232 [1.027, 1.477]	0.51	**0.025** (*)
LB DS	0.938 [0.750, 1.173]	0.85	0.575
FL DS	0.993 [0.785, 1.255]	0.04	0.951
PM DS	1.416 [1.142, 1.758]	0.03	**0.002** (**)
EM DS	1.140 [0.985, 1.321]	1.16	0.080
amp(1q)	1.764 [1.022, 3.044]	1.26	**0.042** (*)
del(13q)	1.554 [0.955, 2.530]	3.40	0.076
del(17p)	0.393 [0.102, 1.522]	3.02	0.177
amp(1q)×del(13q)	0.670 [0.312, 1.437]	5.82	0.304
PI-containing	0.639 [0.329, 1.242]	—	0.187

Supplementary Table 9: Associative analysis of prognostic factors for PFS adding PI-containing treatment binary covariate. An unpenalised multivariable Cox proportional hazards model was fitted on the full cohort (n = 165, 99 events). Hazard ratios (HR) with 95% confidence intervals and Wald test p-values are reported for each covariate. Continuous and ordinal variables were standardised prior to fitting. Significance notation: *** $p < 0.001$; ** $p < 0.01$; * $p < 0.05$. Significant p-values are in bold

S10 Comparison with R-ISS staging

R-ISS stage was available for 131/165 patients (79.4%). To benchmark the proposed multi-modal framework against established clinical staging, the penalised Cox model was refitted on this subset using the same nested cross-validation framework as the primary analysis (10-fold outer, 5 repetitions). The model achieved a C-index of 0.697 [0.665, 0.728] and an IBS of 0.176 [0.168, 0.184], compared to a C-index of 0.624 [0.595, 0.652] for R-ISS used as a sole ordinal predictor. The improvement in discrimination (ΔC-index = +0.073) suggests that site-specific DS, combined with CNA and clinical variables, captures prognostic information beyond what is provided by R-ISS staging alone. To visualise the relationship between R-ISS and fully automated DS, Sankey diagrams mapping R-ISS stages to BM DS (panels a, b) and LB DS (panels c, d) are shown in Supplementary Fig. 7, for both automated and manually assigned DS. Several observations emerge. First, patients within the same R-ISS stage are distributed across multiple DS categories, indicating that DS captures additional heterogeneity not reflected in clinical staging. This is particularly evident for patients categorised as intermediate R-ISS 2, the largest (n=72) and most heterogeneous group, whose members span DS 2 through DS 5 for both BM and LB. Second, R-ISS 3 patients tend to concentrate in higher DS categories (DS 4-5), consistent with the expected association between advanced staging and higher metabolic activity. Third, comparing automated (panels a, c) with manually assigned DS (panels b, d), the overall flow patterns are broadly consistent; notably, for BM DS, automated scoring assigns fewer R-ISS 3 patients to the lower DS 2-3 categories compared to manual scoring, suggesting a potentially more conservative classification of diffuse marrow involvement by the automated pipeline.

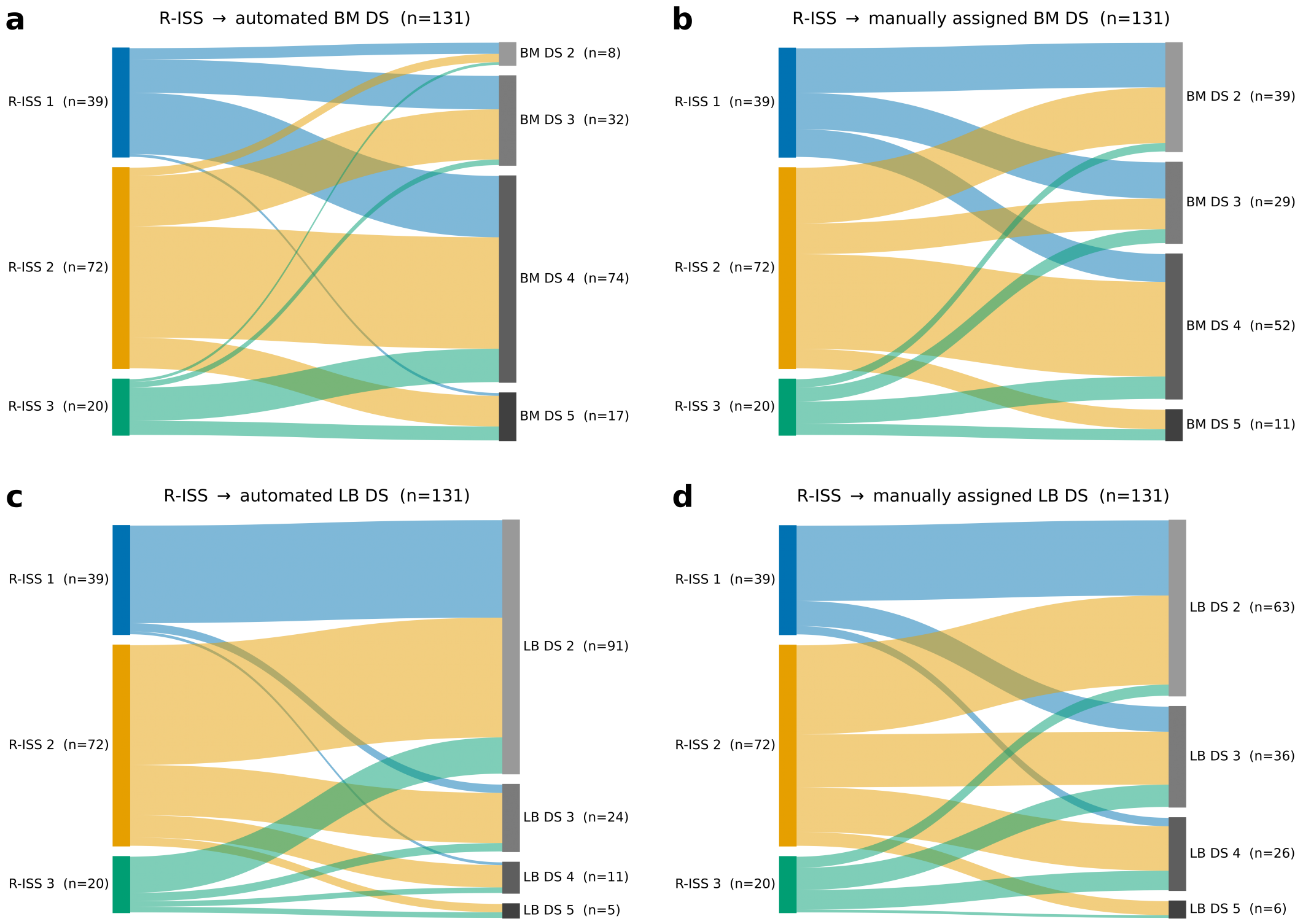

Supplementary Figure 7: Sankey diagrams mapping R-ISS stage to Deauville Score categories for the 131 patients with available R-ISS data. **a** R-ISS to automated BM DS; **b** R-ISS to manually assigned BM DS; **c** R-ISS to automated LB DS; **d** R-ISS to manually assigned LB DS. Flow widths are proportional to the number of patients. Group sizes are indicated in parentheses

S11 Sensitivity analysis on rare events

To assess whether the inclusion of covariates with sparse data affects the estimates of the remaining prognostic factors, the unpenalised multivariable Cox model was refitted after removing del(17p) (11 carriers, 7%) and EM DS (10 patients with DS ≥ 2, 6%) from the covariate set. Both variables yielded potentially unstable HR estimates in the primary analysis due to the small number of informative cases. The resulting model included 11 covariates fitted on the full cohort (n = 165, 99 PFS events). Results are reported in Supplementary Table 10; significance and direction of all remaining covariates were unchanged, confirming that the unstable del(17p) estimate does not affect primary findings.

Covariate	HR [95% CI]	p-value
Age	1.696 [1.364, 2.110]	< **0.001** (***)
Sex	1.462 [0.910, 2.350]	0.117
HB	0.634 [0.506, 0.809]	< **0.001** (***)
PLT	0.934 [0.765, 1.140]	0.502
BM DS	1.189 [0.987, 1.432]	0.068
LB DS	0.932 [0.742, 1.171]	0.546
FL DS	0.995 [0.789, 1.255]	0.967
PM DS	1.333 [1.062, 1.673]	**0.013** (*)
amp(1q)	1.859 [1.066, 3.241]	**0.029** (*)
del(13q)	1.526 [0.946, 2.459]	0.083
amp(1q)×del(13q)	0.574 [0.265, 1.244]	0.160

Supplementary Table 10: Associative analysis of prognostic factors for PFS excluding rare events (EM DS, del(17p)). An unpenalised multivariable Cox proportional hazards model was fitted on the full cohort (n = 165, 99 events). Hazard ratios (HR) with 95% confidence intervals and p-values reported for each covariate. Continuous and ordinal variables standardised prior to fitting. Significance notation: *** $p < 0.001$; ** $p < 0.01$; * $p < 0.05$. Significant p-values are in bold

References

[1] M. Baysal, U. Demirci, E. Umit, H. O. Kirkizlar, E. I. Atli, H. Gurkan, S. K. Gulsaran, V. Bas, C. Mail, and A. M. Demir. Concepts of Double Hit and Triple Hit Disease in Multiple Myeloma, Entity and Prognostic Significance. *Scientific Reports*, 10:5991, 2020.

[2] A. Panopoulou, S. Easdale, M. Ethell, E. Nicholson, M. Potter, A. Giotas, H. Woods, T. Thornton, C. Pawlyn, K. D. Boyd, and M. F. Kaiser. Impact of Ultra High-risk Genetics on Real-world Outcomes of Transplant-eligible Multiple Myeloma Patients. *HemaSphere*, 7:e831, 2023.

[3] C. Nanni, A. Versari, S. Chauvie, E. Bertone, A. Bianchi, M. Rensi, M. Bellò, A. Gallamini, F. Patriarca, F. Gay, B. Gamberi, P. Ghedini, M. Cavo, S. Fanti, and E. Zamagni. Interpretation criteria for FDG PET/CT in multiple myeloma (IMPeTUs): final results. IMPeTUs (Italian myeloma criteria for PET USe). *European Journal of Nuclear Medicine and Molecular Imaging*, 45:712–719, 2018.
